# Function of epithelial stem cell in the repair of alveolar injury

**DOI:** 10.1186/s13287-022-02847-7

**Published:** 2022-04-27

**Authors:** Manwai Chan, Yuru Liu

**Affiliations:** 1 Department of Biomedical Engineering, University of Illinois College of Medicine, Chicago, IL 60612 USA; 2 Department of Pharmacology and Regenerative Medicine, University of Illinois College of Medicine, Chicago, IL 60612 USA; 3 University of Illinois Cancer Center, Chicago, IL60612 USA

**Keywords:** Lung, Alveoli, Type II cells, Type I cells, Progenitor cells, Stem cells

## Abstract

Alveoli are the functional units of blood-gas exchange in the lung and thus are constantly exposed to outside environments and frequently encounter pathogens, particles and other harmful substances. For example, the alveolar epithelium is one of the primary targets of the SARS-CoV-2 virus that causes COVID-19 lung disease. Therefore, it is essential to understand the cellular and molecular mechanisms by which the integrity of alveoli epithelial barrier is maintained. Alveolar epithelium comprises two cell types: alveolar type I cells (AT1) and alveolar type II cells (AT2). AT2s have been shown to function as tissue stem cells that repair the injured alveoli epithelium. Recent studies indicate that AT1s and subgroups of proximal airway epithelial cells can also participate alveolar repair process through their intrinsic plasticity. This review discussed the potential mechanisms that drive the reparative behaviors of AT2, AT1 and some proximal cells in responses to injury and how an abnormal repair contributes to some pathological conditions.

## Background

The mammalian lung consists of a tree-like airway compartment that allows transport of air and millions of honeycomb-like structures called alveoli that serve as the blood-gas-exchanging units [1]. Two types of cells line the alveolar epithelium: alveolar type I cells (AT1) and alveolar type II cells (AT2) [1]. AT1s cover > 95% of the surface area of the alveolar barrier. With their squamous shape, thin and extended surface, these cells align closely with lung microvascular endothelial cells, thus providing the interface for the blood-gas exchange [1]. AT2s are cuboidal cells that predominantly reside at the corner of alveoli. Even though the numbers of AT2s are about twice as many as that of AT1s [2], they cover only < 5% of the alveoli surface area [3]. AT2s are responsible for the secretion of surfactants to keep the surface tension of alveoli. They also play other essential roles such as transportation of ions and fluids and modulation of lung immune responses [3]. AT2s can be identified by expressions of specific markers such as Sftpc, ABCA3, presence of specific intracellular structure such as lamellar body, ability to produce surfactant and their functions described above [4].

Alveoli epithelial barrier is exposed to the external environment thus is constantly bombarded by various pathogens and particles [5]. This is especially important during the current COVID-19 pandemic because alveolar epithelial cells are one of the major targets of SARS-CoV-2, the viral pathogen of the COVID-19 [6–9]. The coronavirus SARS-CoV-2 enters AT2s through an angiotensin-converting enzyme 2 (ACE2) molecule located on the surface of the AT2s [7, 10]. To deal with these damages, alveoli epithelial cells also bear repair and regeneration potentials. Based on studies using in vitro culture, in vivo mouse lineage tracing and injury models, it is well established that AT2s act as progenitor or stem cells in lung repair/regeneration by self-renewal and differentiating into AT1s [11–17].

Recent studies indicate that AT1s possess certain degree of plasticity thus may also play a role in the repair/regeneration [18]. Furthermore, some subgroups of airway epithelial cells can migrate into the alveolar region upon injury and participate in the repair processes [19]. This review will discuss the progenitor cell function of AT2s, including the subgroup of cells engaged in repair, potential regulatory niches, and the intrinsic signals that drive quiescent AT2s to enter a regenerative program. Furthermore, we will discuss the context-dependent plasticity and reparative responses of AT1s and proximal epithelial cells in different developmental stages and different types of injury.

## Main text

### Mechanisms underlining AT2 facultative stem cells-mediated lung repair

**Fig. 1:**
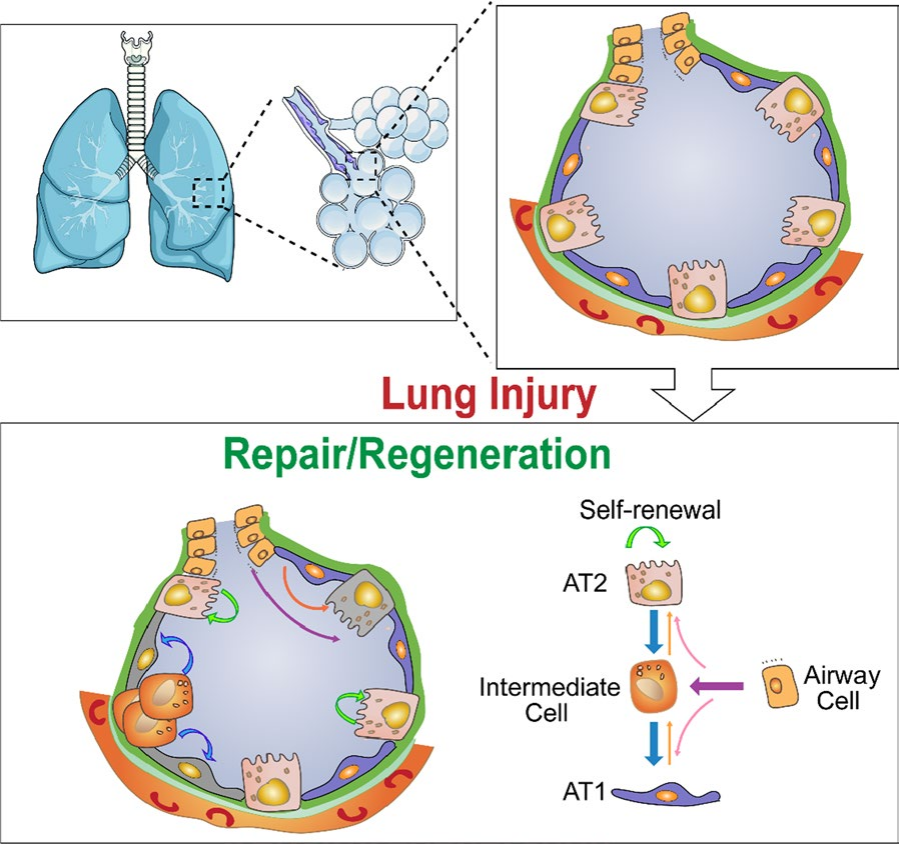
A Model: After lung injury, some AT2s are activated and behave as facultative stem cells. A fraction of the activated AT2s differentiates into intermediate cells in the middle of AT2 to AT1 transition. Many of these transient intermediate cells differentiate into AT1s and repair the alveoli subsequently. Depending on the age and type of injury, sometime AT1s can differentiate into AT2s. After some severe lung injuries, subgroups of airway cells can be mobilized and migrate into alveoli. These cells became certain intermediate cell types and might also be able to transit into AT2s or AT1s

AT2s are normally quiescent with a slow turnover rate, but they can be activated in response to injury and acquire remarkable regenerative capacity [12, 13, 20]. Those AT2s that exit the quiescence and enter the repair program are thus called “facultative stem cells” [21]. For example, in the repair phase after bacteria (*Pseudomonas aeruginosa)*-induced lung injury, 30–70% of the AT2s acquire the expression of a stem cell surface marker Sca-1 (stem cell antigen 1, or Ly6a) [15, 16]. The Sca-1^+^ AT2s are likely to be the cells engaged in repair because they have a relatively higher potential to proliferate and differentiate into AT1s than the rest of AT2s [15, 16]. Further studies indicate that some AT2s enter a stepwise repair program after injury, including proliferation, partial de-differentiation, transition into an AT2-AT1 intermediate state, and finally differentiate into AT1s [15, 16] (Fig. 1).

The recent development of the single-cell RNA-sequencing (scRNA-seq) technique provided a unique tool to characterize these AT2-derived intermediate cells that transiently appear during distinct reparative stages; these studies have resulted in several reports. For example, after LPS-induced mouse lung injury, three AT2 subgroups were detected. Their transcriptome profiles indicate that they are undergoing distinct repair steps: cell proliferation, cell-cycle arrest and trans-differentiation into AT1 [22]. Transient AT2 subpopulations that appear in the middle of AT2 to AT1 transition were also found during lung regeneration in mouse pneumonectomy model [23], during the repair of bleomycin-induced lung injury [24–27], and in the 3D culture of AT2s in which they form alveoli-like organoid [25]. These intermediate cells were initially named by various groups that identified them as “Alveolar Differentiation Intermediate (ADI)” [26], “Pre-Alveolar Type-1 Transitional Cell State (PATS)” [25] or “Damage-Associated Transition Progenitors (DATPs)” [24], respectively; however, the transcriptome of these cells indicates that ADI, PATS, DATP cells as well as the “cell cycle arrest subpopulation” cells are overlapping populations [22]. Lineage tracing analysis and RNA velocity studies supported that these intermediate cells are primarily derived from AT2s and are undergoing differentiation toward AT1s [24–27]. Consistently, these cells express low levels of transcripts considered as AT2 and AT1 markers. Furthermore, several specific markers of these intermediate cells were identified by scRNA-seq: Most of these cells express Krt8 [26]; a fraction of these cells also expressed *Cldn4*, *Krt19*, *Ctgf* and *Sfn* [25]. These cells are rarely detectable in uninjured lungs, but their numbers significantly increase after various types of lung injuries [26]. Furthermore, the aforementioned Sca-1^+^ AT2 cells that appear after pseudomonas-induced lung injury also express higher levels of *Krt8* and *Cldn4* [16] and the ADI cells also showed higher expression of Sca-1 (Ly6a) [26], and thus, it is likely that the Sca-1^+^ AT2s enriched some of these intermediate populations.

Several signaling pathways have been shown to function in various aspects of the AT2-mediated repair process. For example, growth factors FGF and EGF can promote AT2 proliferation [12, 28]. In contrast, BMP4 signaling inhibits AT2 self-renewal but promotes AT2 to AT1 transition [29]. Furthermore, a biphasic temporally regulated Notch activity is required for AT2 to AT1 transition. At the early phase of repair, AT2 Notch activity is elevated, and the high Notch activity is required for the survival of AT2s when these cells become the intermediate cells toward AT1 transition [17]. However, at the later phase, AT2 Notch activity needs to be downregulated by the non-canonical Notch ligand Dlk1 (delta-like 1) so that these cells can further differentiate into AT1s. A constitutively active Notch signaling in AT2s resulted in their blockage in the intermediate stage in the middle of AT2 to AT1 transition [17]. Wnt signaling also plays a vital role in AT2 stem cell function. It has been shown that a subgroup of lineage-traced Wnt-responsive Axin2^+^ AT2s are actively engaged in the lung repair [21, 30]. Wnt signaling appears to be required for the initiation of the repair process. However, subsequent downregulation of β-catenin, a central mediator of the canonical Wnt signaling at a later stage, is needed to complete AT2 to AT1 transition [30]. Furthermore, the Hippo pathway plays an essential role in the transition of AT2 to AT1 as disruption of the YAP signaling blocked this transition [31, 32]. YAP, the central transcription regulator of the Hippo pathway, is typically located at the cytosol of AT2s; but when AT2s start to convert into AT1s, YAP molecules translocate into the nucleus and then remain in the nucleus of AT1s [31, 32]. In addition to the above-mentioned embryogenesis-related signaling, some immunity-related signaling molecules can also regulate AT2 progenitor functions. For example, Toll-like receptor 4 (TLR4) appears to be required for AT2 proliferation after bleomycin-induced injury [33]. Likely downstream to these signaling pathways, several transcription factors, FoxM1 [15], HIF1α [34] and Etv5 [35], have been identified to play critical roles in regulating the functions of reparative AT2s. Furthermore, the aforementioned scRNA-seq studies of AT2-AT1 intermediate cells have uncovered several molecular pathways activated in AT2s during alveolar repair: P53, TGFβ and cell senescence pathways [22, 24–27]. Finally, one of the major functions of AT2 is production of surfactant. A dysregulation of surfactant production can result in defective lung repair after injury, likely due to impair AT2 progenitor functions [36, 37].

**Fig. 2:**
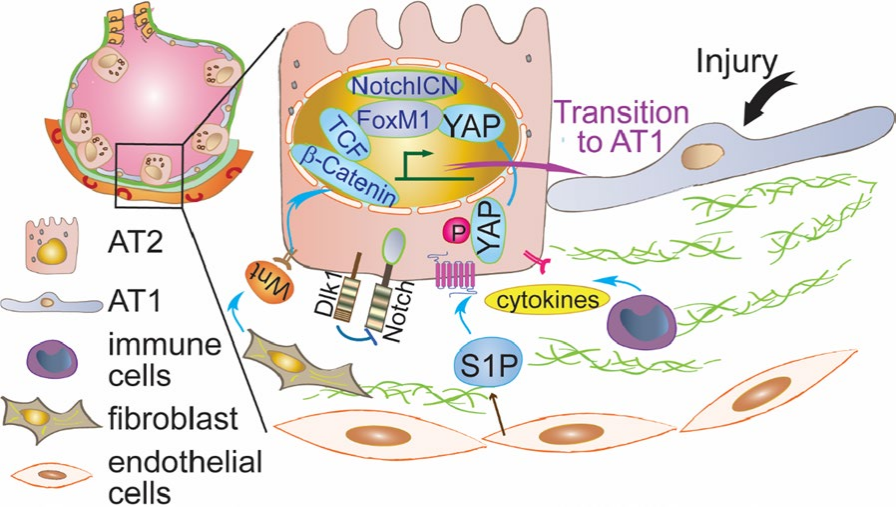
Some examples of the signaling mechanisms involved in AT2 to AT1 transition. Endothelial cells, fibroblasts and immune cells compose the niche that release signals to activate AT2s. These signaling molecules activate their respective receptors expressed on the surface of AT2s. Downstream factors such as YAP, and β-Catenin/TCF are activated to promote the AT2 to become AT2/AT1 transitional cells and finally convert to AT1s. A dynamically regulated Notch by Dlk1 is also needed for AT2/AT1 transition

The functions of AT2 facultative stem cells are regulated by nearby microenvironmental niche cells that can sense the injury. Those niche cells include endothelial cells [38, 39], immune cells [40] and fibroblasts [13, 41]. For example, the activation of YAP in AT2 is likely initiated through cell surface receptor S1PR2, which is triggered by the increased production of interstitial S1P (sphingosine 1 phosphate) by nearby endothelial cells in response to injury [42]. Other than S1P, endothelial cells also serve as niche cells by producing HGF or THBS1 [38, 39]. Fibroblasts have been shown to release niche signals such as PDGFα or Wnts to promote AT2 proliferation and differentiation [13, 30, 41]. Stat3, a signaling molecule previously known to be involved in inflammatory responses, is activated in AT2s and promotes repair by modulating the surrounding fibroblast niche through reciprocal paracrine molecules [43]. Immune cells can also function as niche components [40]. Some inflammatory paracrine factors such as TNF and IL1β released by immune cells recruited after injury also participate in the AT2 reparative programs [24, 40]. Some of these factors may promote AT2 proliferation [40], and the IL1β-mediated activation of the IL1R1 receptor expressed on AT2s might also be involved in the transition of AT2s to intermediate cells, which will further convert to AT1s [24] (Fig. 2).

### The underappreciated role of AT1 in alveolar repair

Even though there are fewer AT1s than AT2s [1, 44, 45], they cover > 95% of the lung surface area due to their large, thin and squamous shape [44, 45] and thus serve as the interface for O_2_-CO_2_ exchange [45]. AT1s have not been studied as thoroughly as AT2 partly because there is a lack of consistent and practical approaches to isolate viable AT1 cells from the lung for in vitro studies. In addition, the shapes of individual AT1 are usually irregular and span several alveoli in a 3D structure, thus making it challenging to observe. However, with the recent development of state-of-the-art techniques such as lineage tracing, 3D imaging reconstruction and scRNA-seq, more knowledge has been obtained about AT1.

AT1 was believed to be a terminally differentiated and quiescent cell type that functions in the exchanges of gas, ion and water, but recent studies suggest that AT1s exhibit some degree of plasticity [18, 46]. Under certain in vitro culture conditions, AT1s can proliferate [18, 46] and differentiate into AT2-like cells [18, 46] and form colonies with alveoli-like structures [18]. The plasticity of AT1s seems to decrease from postnatal stages to adulthood [47]. During the late phase of lung development, i.e., the alveoli maturation stage, AT1s undergo extensive shape changes, including flattening, folding and spreading to cover the large surface area of the alveoli [47, 48]. In addition, AT1s, through secreting various paracrine ligand molecules, serve as “signaling hubs” to direct and coordinate the development of underlining myofibroblasts and microvascular endothelial cells [49].

AT1 cells display different degrees of plasticity during regeneration depending on the animal's age, as well as nature and extent of the injury. A recent study showed that after hyperoxia or hypoxia-induced lung damages in new-born mice, AT2s had limited regeneration capacity [50]; in contrast, lineage-tracing studies using AT1 marker HopX (HOP homeobox) and Ager (Advanced Glycosylation End-Product Specific Receptor) revealed that AT1 cells exhibited robust plasticity in reprogramming itself into AT2 cells [50]. In adult mouse lungs after hyperoxia, AT1s as lineage traced by HopX also appear to be able to convert into AT2s [50]. During alveoli regeneration after partial pneumonectomy (PNX), different results about AT1 plasticity were observed depending on the markers used for lineage tracing. When using the HopX marker, it is found that AT1s can proliferate and reprogram themselves into AT2 cells and thus may replenish the AT2 progenitor pool [18]. When using another AT1 marker, IGFBP2 (insulin-like growth factor-binding protein 2) for lineage tracing, AT1s did not differentiate into AT2 [47]. IGFBP2 appears to be a marker of mature AT1s because the percentage of IGFBP2-expressing AT1s in mouse lung gradually increase after birth from 20% in neonatal to 95% in adults [47]. Thus, it is likely that the adult AT1 cell population contains two distinct subtypes: HopX^+^Igfbp2^+^ and HopX^+^Igfbp2^−^ AT1 cells. HopX^+^Igfbp2^+^ AT1 cells represent most adult AT1s and are terminally differentiated. They cannot transdifferentiate into AT2s and cannot proliferate during alveolar regeneration. In contrast, HopX^+^Igfbp2^−^ cells are fewer in the adult AT1 population (5%), maintain cellular plasticity and participate in repair/regeneration by transition into AT2s [47].

The signaling mechanisms that control AT1 plasticity are largely unknown. Recently, it has been shown that YAP localized in the AT1 nucleus is essential for maintaining the fate of this cell type, as disrupting YAP resulted in the conversion of AT1 into AT2 [50]. It is also unknown whether AT1s can respond to lung injuries other than differentiating into AT2s, e.g., whether AT1s also undergo migration or shape changes that could affect the restoration of the alveolar epithelial barrier.

### Subgroup of airway cells that participate in alveolar repair

In response to some severe lung injuries, specific subpopulations of airway epithelial cells can be mobilized and migrate into the alveolar parenchyma to participate in the repair process. Several reports have shown that after H1N1 influenza viral infection, airway-derived cells became Trp63^+^/Krt5^+^, migrated into the alveoli region and form “pods.” These pods appear to be a temporal cellular structure that can seal the denuded alveolar barrier [51–53]. These airway cells were named “distal airway stem cells” (DASC) [53] or “lineage-negative epithelial progenitors” (LNEPs) [51] by independent groups that discovered them. Further lineage tracing analysis showed that these cells are derived from Sox2^+^ airway epithelial progenitor cells [54].

The potential for these cells to give rise to AT2 and AT1 cells during repair is unclear, and the signaling mechanisms regulating these cells' regenerative responses are incompletely understood. One study showed that the initial activation of Trp63^+^/Krt5^+^ cell requires Notch signaling, whereas subsequent blockade of Notch signaling in these cells can induce them to differentiate further and express AT2 marker Sftpc [51]. In addition, HIF1α protein that senses local hypoxia can coordinate with Notch and regulate the activation of Trp63^+^/Krt5^+^ cells [55]. FGF signaling also promotes the regenerative function of these cells [56]. Further studies showed that during alveolar repair, some LNEP cells, especially those derived from a subpopulation that expresses a higher level of antigen-presenting protein H2-K1, can also form transient cells populations that are similar to the above described Krt8^+^ AT2/AT1 intermediate cells [26, 52], but it is unclear to what extent will these Krt8^+^ cells further become AT2s or AT1s and whether the airway cell-derived alveolar cells are distinct from endogenous AT2s and AT1s.

Some other airway cells also possess certain cellular plasticity so that they may participate in alveolar repair. A recent report showed that some secretory cells could be activated by adjacent ciliated cells through IL1β and notch signaling and further differentiate into AT2-like cells [57]. Another group of airway-derived stem cells is named bronchioalveolar stem cells (BASCs) [58]. These cells mainly reside at the bronchioalveolar duct junction (BADJ), expressing the AT2 marker Sftpc and airway Club cell marker Scgb1a1. Lineage tracing studies showed that BASCs could differentiate into bronchiolar and alveolar epithelial cells [38, 59, 60]. However, the extent to which BASCs contribute to alveolar repair is hard to determine because these cells cannot be distinguished by lineage tracing with some Scg1b1a expressing AT2 cells that constitute around 10% of the total AT2s and reside in the peripheral alveoli region away from the BADJ [61].

### Implication of impaired alveolar repair in lung disease

The impaired alveolar repair could result in chronic lung diseases such as cancer, fibrosis and emphysema [62]. Even though the detailed mechanisms for the pathological changes in these diseases are still unclear, recent studies indicate that some of the transient cell types that appeared during the repair/regeneration process may be responsible for the onset of these diseases. For example, the aforementioned AT2-AT1 intermediate cells are rarely detected in normal lungs [26, 63, 64], but increased number of cells expressing the markers of this intermediate population, such as KRT8, CLDN4 and SFN, were detected in patient lungs of idiopathic pulmonary fibrosis (IPF) [24–26], and recent studies indicate that these aberrant intermediate cells might activate the surroundings fibroblasts and contribute to the formation of fibrosis [23, 65]. Consistently, scRNA-seq studies have revealed that the epithelium of normal human lungs is composed of mature differentiated cell types, e.g., AT1s or AT2s, whereas in the lung of IPF (idiopathic pulmonary fibrosis) patients, various atypical epithelial cell subgroups were identified. Those cells showed co-expression of markers of AT1s, AT2s and airway cells; this indicates that these cells are the results of aberrant differentiation that were trapped in certain intermediate stages [66–69]. Furthermore, the migration of airway-derived epithelial cells into the alveolar region may result in the proximalization of alveoli which is also a hallmark of IPF [51]. Similar abnormal cells are also present in the lungs of COPD patients [66]. Moreover, abnormalities of some of the signaling molecules and transcription factors that regulate the functions of these intermediate cells may be related to the progression of fibrosis; for example, YAP, TGF-β, P53 and WNT are implicated in the pulmonary fibrosis formation [66–69]. Persistent Notch activity can disrupt airway stem cell-mediated repair and result in the generation of abnormal cysts structure resembling the honeycomb formation in fibrosis patients [51].

AT2 is a main origin of lung adenocarcinoma [70]. The repeated injury and impaired repair may also contribute to the neoplastic transformation of AT2s. We believe in most case after injury, AT2 proliferation is regenerative response. However, it is also possible that uncontrolled AT2 proliferation can be pathological and may lead to hyperplasia and later to adenocarcinoma. In fact, cells expressing markers of AT2/AT1 intermediate cells also appear in lung adenocarcinoma samples [24], and this suggests that the repair response of AT2 may be related to carcinogenesis processes.

In lungs with COVID-19, AT2s have reduced expression of normal AT2 marker Sftpc [71], whereas there is increased number of the cells that express markers of AT2/AT1 intermediates [72]. Thus, knowledge about AT2 stem/progenitor cell function will lead to better understanding of the pathogenesis and recovery of COVID-19. Other than examining the patient samples, the recently developed iPSC-derived AT2 and organoid culture system has provided valuable tools to identify the pathological response in AT2 after SARS-CoV-2 infection as well as screening for potential drugs to treat this disease [73–75].

## Conclusions

Lung epithelial stem cells are different from some other tissue stem cells in organs of high turnover rate such as bone marrow, skin or intestine. In those organs, certain groups of stem cells usually reside at a specific anatomic location and these specific cells are responsible for the tissue homeostasis and repair [76]. In contrast, in the lung several kinds of mature cells such as AT2s can be activated upon injury to behave as facultative stem cells. Distinct subpopulations of epithelial cells possess various degrees of plasticity so that multiple cell types including AT2s, AT1s and airway cells can undergo trans-differentiation and contribute to the repopulation of denuded alveolar barrier (Fig. 1).

A critical feature of these facultative stem cells involved in lung repair is their plasticity resulting in cell type transition, e.g., AT2 to AT1, AT1 to AT2, airway epithelial cells to alveolar epithelial cells. These transitions generate several kinds of intermediate cells that likely contribute to various disease states. The recent advance of state-of-the-art techniques such as lineage tracing and scRNA-seq studies has allowed people to characterize such intermediate cells. However, many outstanding questions remain and to answer them, we would need further improvement of these technologies. For example, dual or multi-markers lineage tracing technique [59, 60] will be required to study various cell subpopulations. Improvement on the scRNA-seq technique is also needed for a more in-depth analysis of the regulatory genes products, which usually have low expression levels. For further studies, combined approaches such as mouse genetic mutant model, cell isolation using surface markers and cell transplantation will be needed to interrogate the heterogeneous intermediate subpopulations of cells that appear during repair and in lung diseases. Furthermore, most of our knowledge regarding alveolar repair is obtained using mouse models [19, 77]. However, significant differences exist between mouse and human lungs and airways; for instance, human airways can be divided into two anatomical components: conducting airways and respiratory airways, whereas respiratory airways have not been found in mice [78]. Therefore, studies using human tissues and cells such as iPSC-derived lung organoids [74] and precision-cut lung slices (PCLS) [79] are necessary to elucidate human-specific mechanisms in alveolar repair.

## Data Availability

Not applicable.

## Abbreviations

AT1: Alveolar type I cell
AT2: Alveolar type II cell
COVID-19: Coronavirus disease 2019
ACE2: Angiotensin-converting enzyme 2
scRNA-seq: Single-cell RNA-sequencing
BMP: Bone morphogenetic protein
YAP: Yes-associated protein
TNF: Tumor necrosis factor
HIF: Hypoxia-inducible factor
KGF: Keratinocyte growth factor
BASC: Bronchioalveolar stem cells
ARDS: Acute respiratory distress syndrome
COPD: Chronic obstructive pulmonary disease
IPF: Idiopathic pulmonary fibrosis
